# The Sense of Death

**Published:** 1908-04-18

**Authors:** 


### The Sense of Death.

A circular issued recently by the Local Government Board on the subject of the humane slaughtering of animals brings to mind again a difficult question which every man has to settle with his own conscience—namely, the ethics of killing. The difficulty arises in the main from the need for compromise on the one hand, and, on the other, from the obstacles to a consistent attitude which such a compromise involves. To the Buddhist his course is clear: he may not take life at all. To the savage also his course is clear, for his right to kill what (and whom) he will is limited only by his ability to do the killing. But a people which recognises the right of mankind to take the life of the lower animals while it remains susceptible to motives of humanity and sympathy cannot, in the nature of things, escape twinges of conscience from time to time. These twinges, of course, assail most severely those members of the complex body-politic who are blessed, or cursed, with the highest sensibility, and the response to the stimulus is seen in the creation of an anti-vivisectionist, a conscientious vegetarian, or a member of the S.P.C.A., according to the temperamental endowments of the individual concerned. These three types represent "No compromise" in a descending scale of thoroughness, the last merging insensibly with the general mass of our society, a mass with a leaning towards kindliness, easily aroused to indignation by an act of gross cruelty, but willing, nevertheless, in judging the infliction of pain upon animals, to consider the value of the end achieved. This class, realising the impossibility of attaining a strictly logical rule of conduct, prefers not to dwell upon the theme. It is actuated by certain large rules of right and wrong in the business, the fruit of education and environment, and may be typified by the man who dismounts and takes off his coat to chastise the carman who is ill-treating his horse, and forthwith mounts again to ride a fox to death. The latter proceeding has been sanctified by custom, and does not transgress his simple code of ethics. If he were questioned on the matter one can imagine his saying that the price of a fox's life is more than counterpoised by the development of hardihood and other manly qualities which result in the followers of hounds. But the ill-treatment of a horse indicates an explosion of anger or malice, and must be reprehended. It is true he kills the fox, or at least abets the murder, but he kills it as though he loves it, not from any motive of personal pique. But to the "No-compromiser" such casuistry is unpardonable. None the less, the more rigidly he defines his rule of conduct the harder it is not to offend it grossly, and we have yet to hear of the conscientious vegetarian who has forsworn the wearing of boot leather because it postulates an unjustifiable killing.

The impossibility of maintaining a logical attitude in this matter does not, however, excuse us from not considering it. Such contemplation promotes peace of mind, for it undoubtedly substantiates the worn Shakespearian tag, "The poor beetle that we tread upon in corporal sufferance finds a pang as great as when a giant dies." The "apprehension" upon which the poet lays such stress as being the chief part of "the sense of death," and which none but the most imaginative of humanitarians concedes to the lower animals, is undoubtedly the cardinal element in the ethical problem; and very comforting it is to feel that this apprehension is the hard prerogative of man. The death-bed experience of our profession is enough to tell us that the act of dissolution is seldom in itself attended by much pain, while those who may almost be said to have experienced death by violence, in the sense that they have been within an ace of death by bullet-wound or accident, lay little stress upon the pang of the moment. If this be true, as we believe it to be, we must credit the attitude of the advanced humanitarians to an anthropomorphic conception of the lower creation which is unjustified by the probabilities of the case. Such a conception is expressed in an advertisement of the anti-vivisectors which at this moment adorns the walls of certain railway-lifts in this city—a dog with speaking eyes begging to the monster who, we are to suppose, is about to perform upon it. This kind of thing may be good campaigning, but we believe it to be bad comparative psychology and a purely emotional appeal to popular ignorance and prejudice. It is idle to attempt to convince the advanced school of "No-compromise" on a point of ethics which is admittedly arguable; but we are happy to think that the saner majority is still capable of tempering the instinctive humanity of the Briton with some sense of proportion, and some appreciation of the difference in mental equipment which both for better and for worse has given man his pre-eminence in the animal kingdom.

